# The association between global and prime diet quality scores and the odds of metabolic-associated fatty liver disease: a case-control study

**DOI:** 10.3389/fnut.2025.1664091

**Published:** 2025-10-24

**Authors:** Georgios Zacharakis, Hanan Alyami, Tariq Alrasheed, Naif S. Almutairi, Gaber Mohamed Gomaa Shehab, Mohamed Goda Elbqry, Majid Ali Alotni

**Affiliations:** 1 Department of Internal Medicine College of Medicine, Prince Sattam bin Abdulaziz University, Al-Kharj, Saudi Arabia; 2 Medical and Surgical Nursing, Nursing College, Princess Nourah bint Abdulrahman University, Riyadh, Saudi Arabia; 3 Department of Internal Medicine, University of Tabuk, Tabuk, Saudi Arabia; 4 Faculty of Health Sciences, Umm Al-Qura University, Makkah, Saudi Arabia; 5 Department of Health Management and Medical Information, College of Health Sciences at Al-Leith, Umm Al-Qura University, Al-Leith, Saudi Arabia; 6 Biochemistry Department, College of Medicine, Taif University, Taif, Saudi Arabia; 7 Department of Medical and Surgical, College of Nursing, Qassim University, Buraydah, Saudi Arabia; 8 Department of Medical and Surgical Nursing, College of Nursing, Qassim University, Almleda, Saudi Arabia

**Keywords:** metabolic-associated fatty liver disease, MAFLD, global diet quality score, GDQS, prime diet quality score

## Abstract

**Background:**

Metabolic-associated fatty liver disease (MAFLD) is a leading cause of chronic liver disease worldwide, which is closely linked to poor dietary habits, obesity, and metabolic dysfunction. The Global Diet Quality Score (GDQS) and Prime Diet Quality Score (PDQS) are newly developed tools for assessing diet quality across diverse populations. However, evidence on their relationship with MAFLD remains limited. This study aimed to investigate the association between GDQS and PDQS and the odds of MAFLD using a case–control design.

**Methods:**

We conducted a case–control investigation at Prince Sattam bin Abdulaziz University Hospital, Al-Kharj, Saudi Arabia, with participant enrollment from February 2023 to January 2025. The study cohort consisted of 225 cases and 225 controls. Dietary intake was assessed using a semi-quantitative food frequency questionnaire to calculate GDQS and PDQS. Cases and controls were matched by age (±3 years). An unconditional logistic regression analysis was performed to estimate odds ratios (ORs) and 95% confidence intervals (CIs).

**Results:**

Cases had lower GDQS and PDQS compared to controls (*p* < 0.001), with a higher consumption of refined grains and sugar-sweetened beverages and a lower intake of fruits, vegetables, and legumes. Each 1-SD increase in the GDQS and PDQS was associated with approximately 40% lower odds of MAFLD (OR = 0.61; 95% CI: 0.47, 0.79 and OR = 0.60; 95% CI: 0.46, 0.79, respectively).

**Conclusion:**

Higher GDQS and PDQS scores are associated with reduced MAFLD risk, suggesting that improving diet quality could be a key strategy for MAFLD prevention in clinical and public health settings.

## Introduction

Metabolic-associated fatty liver disease (MAFLD) is a leading cause of chronic liver disease globally, driven by obesity, insulin resistance, and metabolic dysfunction (1, 2). Defined by hepatic steatosis with metabolic risk factors, the condition affects approximately 25% of adults worldwide, with higher prevalence in populations with obesity and type 2 diabetes (3, 4). MAFLD arises from complex interactions between genetic, environmental, and lifestyle factors, with diet playing a pivotal role in its onset and progression (5, 6).

Diet, as a modifiable risk factor, plays a critical role in MAFLD development and progression, yet the precise impact of diet quality, particularly in diverse populations, remains underexplored. Emerging evidence suggests that dietary patterns influence MAFLD through mechanisms such as inflammation, oxidative stress, and gut microbiota alterations (7, 8). For instance, a high intake of fructose and saturated fats has been linked to increased liver fat accumulation, while diets rich in fiber and antioxidants may mitigate MAFLD risk (9). However, studies exploring specific dietary components, such as antioxidants, have yielded mixed results (10).

The Global Diet Quality Score (GDQS) and Prime Diet Quality Score (PDQS) are novel tools designed to assess diet quality across diverse populations, capturing both nutrient adequacy and food group consumption (11). These scores have shown promise in predicting non-communicable disease risk, including type 2 diabetes and cardiovascular disease (12). Inconsistent findings highlight the need for further investigation into the nuanced relationship between diet quality and MAFLD, particularly using the standardized Global Diet Quality Score (GDQS) and Prime Diet Quality Score (PDQS). Despite their potential, the application of GDQS and PDQS in MAFLD research remains underexplored, with limited evaluation of their association with disease odds. This study aimed to investigate whether higher GDQS and PDQS are associated with lower odds of MAFLD in a case–control study conducted in Al-Kharj, Saudi Arabia.

## Method

### Study population

This case–control study was conducted at Prince Sattam bin Abdulaziz University Hospital (Al-Kharj, Saudi Arabia) and included adults aged 20–60 years who were referred to the Liver and Gastroenterology Clinic. A total of 450 participants were recruited between February 2023 and January 2025, comprising 225 patients newly diagnosed with metabolic dysfunction-associated fatty liver disease (MAFLD) and 225 age-matched healthy controls. The diagnosis of MAFLD was based on the presence of hepatic steatosis, confirmed by abdominal ultrasonography performed by experienced radiologists. Characteristic ultrasonographic features included hepatorenal echo contrast, vascular blurring, or posterior beam attenuation, together with evidence of metabolic dysfunction. Elevated liver enzymes (ALT > 30 U/L in men, >19 U/L in women; AST > 30 U/L in men, >25 U/L in women) were collected and reported as supportive clinical data but were not used independently as the diagnostic criteria. Metabolic dysfunction was defined as meeting at least one of the following: (1) overweight or obesity (BMI ≥ 23 kg/m^2^, Asian-specific cut-off); (2) type 2 diabetes mellitus (fasting glucose ≥126 mg/dL, HbA1c ≥ 6.5%, or current use of antidiabetic medication); or (3) at least two of the following metabolic risk factors: increased waist circumference (≥90 cm in men, ≥80 cm in women), elevated blood pressure (≥130/85 mmHg), use of antihypertensive medication, high fasting triglycerides (≥150 mg/dL) or lipid-lowering therapy, low HDL cholesterol (<40 mg/dL in men or <50 mg/dL in women), or prediabetes (fasting glucose 100–125 mg/dL or HbA1c 5.7–6.4%). The control group was recruited from hospital visitors who underwent routine health check-ups and had no clinical or ultrasonographic evidence of fatty liver disease. To reduce confounding, controls were frequency-matched to cases by age within ±3 years. The general exclusion criteria for both groups included significant alcohol intake (≥30 g/day for men; ≥20 g/day for women), other chronic liver diseases (viral hepatitis with negative HBsAg and anti-HCV, autoimmune hepatitis, Wilson’s disease, and hemochromatosis), use of steatogenic or hepatotoxic medications, chronic kidney disease, malignancy, thyroid disorders, autoimmune disorders, pregnancy, and medically restricted diets (e.g., for weight loss). Participants completing fewer than 35 items on the food frequency questionnaire or reporting implausible daily energy intake (<800 kcal/day or >4,500 kcal/day) were excluded and replaced. All participants provided written informed consent.

### Dietary intake

The dietary intake of participants was assessed using a validated semi-quantitative food frequency questionnaire (FFQ) that included 152 distinct food items. This tool was designed to capture participants’ habitual dietary patterns over the previous year (13). In accordance with other case–control studies, only newly diagnosed MAFLD cases were enrolled. To minimize potential recall bias and reverse causation, individuals who reported following a specific diet (e.g., weight-loss or therapeutic regimens) or who had recently changed their dietary habits after diagnosis were excluded from the study. Participants were instructed to report their typical dietary habits during the year prior to diagnosis (for cases) or prior to study enrollment (for controls). The FFQ offered a structured set of response options for consumption frequency, ranging from “never or less than once per month” to “six or more times per day.” The reported responses were analyzed using Nutritionist IV software to convert food-frequency data into daily energy and nutrient intakes, including macronutrients, micronutrients, and other bioactive compounds. This approach enabled a robust and comprehensive assessment of usual dietary intake in relation to health outcomes.

Physical activity was assessed using the International Physical Activity Questionnaire (IPAQ). Trained interviewers administered the IPAQ in face-to-face sessions with participants. This validated questionnaire records the frequency and duration of walking, moderate-intensity, and vigorous-intensity activities during a typical day. Each activity was assigned a standard metabolic equivalent (MET) value, and the product of MET-hours/day was calculated. Total physical activity was then expressed as MET-hours/day, following established scoring protocols (14, 15).

### Global diet quality score

To compute the global diet quality score (GDQS), the participants’ daily intake (in grams) of various foods was grouped into 25 distinct food categories. These comprised 16 health-promoting groups—namely, fish and shellfish, poultry and game meats, eggs, low-fat dairy products, whole grains, cruciferous vegetables, dark green leafy vegetables, deep orange vegetables, other vegetables, citrus fruits, deep orange fruits, other fruits, deep orange tubers, legumes, nuts and seeds, and liquid oils; two moderately beneficial food groups—red meats and high-fat dairy; and seven food groups considered detrimental to health—including refined grains and baked goods, white roots and tubers, fruit juices, sugar-sweetened beverages, sweets and ice creams, fried foods, and processed meats. Each group was classified into three or four consumption levels.

Scoring for the health-promoting food groups was as follows:

- A score of 0 was assigned for low intake across all 16 healthy groups.
- Moderate and high consumption of cruciferous vegetables, deep orange vegetables, other vegetables, and deep orange tubers received 0.25 and 0.5 points, respectively.
- The intake of citrus fruits, deep orange fruits, other fruits, whole grains, liquid oils, fish and shellfish, poultry and game meats, and low-fat dairy was scored 1 point for moderate intake and 2 points for high intake.
- For eggs, dark green leafy vegetables, deep orange vegetables, legumes, and nuts and seeds, moderate and high intakes were scored 2 and 4 points, respectively.

For the moderately beneficial (optimal) groups,

- both low and very high consumption levels were scored 0,
- moderate intake was awarded 1 point, and
- high consumption was given 2 points for red meat and high-fat dairy.

Regarding the unhealthy food groups,

- low consumption was awarded 2 points,
- moderate intake received 1 point, and
- high intake was scored 0 for refined grains, white roots and tubers, fruit juices, sugar-sweetened drinks, sweets and ice creams, fried foods, and processed meats.

The final GDQS was derived by summing the points from all 25 food groups, resulting in a total score ranging from 0 to 49 (16).

### Prime diet quality score

The Prime Diet Quality Score (PDQS) is based on the intake of 21 distinct food groups, which are classified into two categories: beneficial (healthy) and detrimental (unhealthy) dietary components. Each of these food groups was initially categorized into tertiles based on consumption levels. For the healthy components—such as low-fat dairy products, poultry, whole grains, fish and shellfish, legumes and soy products, nuts and seeds, vegetable oils, citrus fruits, other fruits, deep orange fruits, cruciferous vegetables, dark leafy greens, deep orange vegetables, and other vegetables—participants were awarded scores as follows: 0 points for the lowest tertile, 1 point for the middle tertile, and 2 points for the highest tertile of intake. In contrast, for unhealthy dietary components—such as processed meats, red meats, sugar-sweetened beverages, refined grains, fried foods, and sweets—the scoring system was reversed: participants received 2 points for the lowest tertile, 1 point for the middle tertile, and 0 points for the highest tertile. The overall PDQS score, derived by summing the scores of all food groups, ranges from 0 to 42, with higher scores reflecting better diet quality (17).

### Statistical analysis

All statistical procedures were carried out using SPSS software (version 23.0; IBM Corp., Chicago, IL, United States). To compare categorical variables between the groups, the chi-squared test was used. For continuous variables, either the independent *t*-test or the Mann–Whitney U test was applied, depending on the distribution of the data. Continuous data are reported as either the median with interquartile range (25th–75th percentile) or mean ± standard deviation (SD), while categorical data are expressed as percentages. To assess the association between GDQS and PDQS and the likelihood of MAFLD, the unconditional logistic regression analysis was conducted. Both unadjusted (crude) and adjusted models were developed, and odds ratios (ORs) along with 95% confidence intervals (CIs) were reported. Food-group comparisons were considered exploratory; therefore, emphasis was placed on reporting effect sizes with 95% confidence intervals rather than relying on statistical significance testing. No adjustment for multiplicity was performed.

## Results

Baseline characteristics of participants differed between the MAFLD and healthy control groups. Statistically significant differences were observed in BMI (*p* = 0.023), family history of MAFLD (*p* < 0.001), and physical activity (*p* = 0.043). Additionally, the case group showed a significantly higher median intake of total fat (*p* = 0.025), whereas dietary fiber intake was notably higher in the control group (*p* = 0.037). These findings are summarized in Table 1.

**Table 1:** Comparison of baseline characteristics and nutrient intake between the control and MAFLD groups in the study population.

Variable	MAFLD (*n* = 225)	Control (*n* = 225)	*p*-value
Baseline characteristics			
Age (year)^a^	32.49 (27.49–35.99)	34.49 (26.49–39.49)	0.341
BMI (kg/m^2^)^a^	29.59 (26.79–32.19)	26.09 (23.69–29.39)	**0.023**
Physical activity (MET/h/day)^a^	26.32 (16.49–46.49)	29.49 (17.49–52.49)	**0.043**
Sex (female) n (%)^b^	118 (52.4)	114 (50.7)	0.158
Familial history of MAFLD, yes, %^b^	113 (50.2%)	55 (24.4%)	**<0.001**
Marital status (married) n (%)^b^	193 (85.8)	199 (88.4)	0.374
Smoking history, yes, %^b^	22 (9.7%)	14 (6.2%)	0.134
Dietary intakes			
Energy (kcal/day)^a^	2483.61 (1721.33–3277.30)	2225.82 (1763.75–3045.04)	0.295
Protein (g/day)^a^	78.65 (61.35–101.61)	84.24 (62.26–113.35)	0.083
Total fat (g/day)^a^	102.40 (70.53–127.35)	85.95 (64.63–110.44)	**0.025**
Carbohydrate (g/day)^a^	328.22 (219.22–451.72)	309.96 (220.26–414.69)	0.146
Fiber (g/day)^a^	20.58 (14.99–29.54)	27.24 (19.32–39.76)	**0.037**

BMI, body mass index; kg, kilogram; m, meter; MET, metabolic equivalent of task; kcal, kilocalorie; g, gram; MAFLD, metabolic-associated fatty liver disease.

a Using the Mann–Whitney U-test, values are presented as median (25th–75th).

b Using the chi-squared tests for categorical variables, values are presented as percentages.Significant values are presented in bold.

Dietary intake from different food groups also varied between the MAFLD and healthy participants. Overall, the mean scores for both the GDQS and PDQS, including their respective components, were significantly higher in the control group compared to those with MAFLD (*p* < 0.05). A closer look at food group consumption revealed that individuals in the control group had significantly higher median intakes of other fruits (*p* = 0.037), dark green leafy vegetables (*p* < 0.001), other vegetables (*p* = 0.002), deep orange tubers (*p* = 0.026), legumes (*p* < 0.001), and poultry/game meats (*p* = 0.041). In contrast, the case group reported greater consumption of refined grains and baked goods (*p* = 0.002), processed meats (*p* = 0.006), sugar-sweetened beverages (*p* < 0.001), and fried foods (*p* = 0.002). These results are presented in Table 2.

**Table 2:** Comparison of food group consumption between healthy individuals and those with MAFLD.

Variable	MAFLD (*n* = 225)	Control (*n* = 225)	Effect size (95% CI)	*P*-value
Positive GDQS score^a^	18.20 ± 4.51	20.55 ± 5.41	*d* = −0.47 (−0.67 to −0.27)	**0.004**
Negative GDQS score^a^	13.47 ± 1.82	14.30 ± 1.86	*d* = −0.45 (−0.65 to −0.25)	**<0.001**
Total GDQS score^a^	27.83 ± 5.08	31.02 ± 5.37	*d* = −0.61 (−0.81 to −0.41)	**0.004**
Healthy PDQS score^a^	16.75 ± 4.63	18.69 ± 5.94	*d* = −0.37 (−0.57 to −0.17)	**<0.001**
Unhealthy PDQS score^a^	12.42 ± 3.14	16.75 ± 4.63	*d* = −1.09 (−1.30 to −0.88)	**0.003**
Total PDQS score^a^	27.28 ± 6.04	33.88 ± 5.11	*d* = −1.18 (−1.39 to −0.97)	**<0.001**
Citrus fruits (g/day)^b^	63.83 (24.91–78.61)	57.16 (30.91–120.58)	*r* = 0.03	0.624
Deep orange fruits (g/day)^b^	76.12 (39.83–114.73)	78.08 (48.23–162.38)	*r* = −0.06	0.196
Other fruits (g/day)^b^	59.93 (33.62–103.31)	87.19 (44.93–162.7)	*r* = −0.14	**0.037**
Dark green leafy vegetables (g/day)^b^	29.18 (15.58–49.29)	49.35 (25.03–86.06)	*r* = −0.27	**<0.001**
Cruciferous vegetables (g/day)^b^	4.58 (3.10–9.43)	7.75 (4.49–16.44)	*r* = −0.07	0.247
Deep orange vegetables (g/day)^b^	7.52 (4.18–13.83)	6.50 (4.73–13.87)	*r* = 0.07	0.201
Other vegetables (g/day)^b^	160.09 (113.75–240.45)	207.71 (122.43–312.53)	*r* = −0.20	**0.002**
Deep orange tubers (g/day)^b^	8.69 (3.96–15.35)	11.13 (7.06–17.90)	*r* = −0.13	**0.026**
Legumes (g/day)^b^	22.47 (11.24–28.42)	33.62 (15.92–74.47)	*r* = −0.24	**<0.001**
Nuts and seeds (g/day)^b^	9.80 (5.84–18.38)	9.57 (5.34–18.11)	*r* = 0.04	0.471
Whole grains (g/day)^b^	5.97 (4.55–7.83)	4.89 (3.99–9.04)	*r* = 0.02	0.751
Refined grains and baked goods (g/day)^b^	425.83 (292.93–609.77)	340.87 (243.83–478.13)	*r* = 0.20	**0.002**
White roots and tubers (g/day)^b^	18.08 (9.53–40.47)	28.25 (9.53–40.47)	*r* = −0.05	0.403
Liquid oils (g/day)^b^	15.33 (8.11–22.63)	15.83 (8.83–21.83)	*r* = −0.04	0.482
Red meats (g/day)^b^	48.41 (24.98–58.14)	50.49 (27.14–88.08)	*r* = −0.08	0.173
Processed meats (g/day)^b^	5.84 (3.83–7.83)	4.54 (3.83–8.17)	*r* = 0.17	**0.006**
Fish and shellfish (g/day)^b^	6.78 (4.83–12.49)	9.34 (5.35–18.21)	*r* = −0.20	**0.002**
Poultry and game meats (g/day)^b^	16.68 (12.40–21.33)	21.97 (12.40–33.83)	*r* = −0.13	**0.041**
Eggs (g/day)^b^	26.25 (12.83–33.14)	26.75 (13.83–42.04)	*r* = −0.08	0.202
Low-fat dairy products (g/day)^b^	228.08 (90.72–292.13)	250.50 (81.17–450.25)	*r* = −0.04	0.466
High-fat dairy products (g/day)^b^	62.16 (25.92–148.35)	52.43 (23.16–138.78)	*r* = 0.09	0.137
Sweets and ice creams (g/day)^b^	53.14 (27.32–92.18)	42.25 (25.13–73.83)	*r* = 0.11	0.078
Sugar-sweetened beverages (g/day)^b^	28.32 (8.54–51.83)	12.99 (8.66–38.83)	*r* = 0.28	**<0.001**
Juices (g/day)^b^	6.54 (3.83–21.66)	7.22 (3.83–20.33)	*r* = −0.05	0.397
Fried foods (g/day)^b^	17.83 (3.83–21.83)	12.73 (3.83–15.33)	*r* = 0.20	**0.002**

GDQS, global diet quality score; PDQS, prime diet quality score; g, gram; MAFLD, metabolic-associated fatty liver disease.

Comparisons are exploratory; effect sizes are shown with 95% CIs. *p*-values are descriptive and not used for formal inference.

a Using an independent sample t-test, values are presented as mean ± SD.

b Using the Mann-Whitney U-test, values are presented as median (25th-75th).Significant values are presented in bold.

The findings from the logistic regression models examining the association between diet quality scores and the odds of MAFLD are presented in Table 3. A strong, graded, inverse association was observed between higher diet quality scores and the odds of MAFLD. In the core confounder-adjusted model (Model B), each 1-standard deviation (SD) increase in the GDQS (OR = 0.61; 95% CI: 0.47, 0.79) and PDQS (OR = 0.60; 95% CI: 0.46, 0.79) was associated with a significantly reduced odds of MAFLD. This protective association was consistently observed across tertiles of consumption. Compared to the lowest tertile (T1), participants in the highest tertile (T3) of GDQS (OR = 0.32; 95% CI: 0.19, 0.55) and PDQS (OR = 0.29; 95% CI: 0.17, 0.51) had approximately 70% lower odds of MAFLD (P-trend <0.001 for both). The association was primarily driven by the intake of healthy food components. A 1-SD increase in the Positive GDQS score (reflecting healthier foods) was associated with a 43% reduction in MAFLD odds (OR = 0.57; 95% CI: 0.44, 0.74). Similarly, the Healthy PDQS component showed a significant inverse association with MAFLD risk in the fully adjusted model (OR per 1-SD = 0.74; 95% CI: 0.59, 0.93). In contrast, the Negative GDQS score, representing less healthy food items, was not significantly associated with MAFLD odds in any model (e.g., Model B OR per 1-SD = 0.89; 95% CI: 0.69, 1.14; P-trend = 0.355). The association for the Unhealthy PDQS component was attenuated and lost statistical significance after adjusting for core confounders, especially after further adjusting for BMI and macronutrients in Model C (P-trend = 0.097).

**Table 3:** Association between tertiles of global and prime diet quality scores and the odds of MAFLD.

Variable	Tertile	Model A OR (95% CI)	Model B OR (95% CI)	Model C OR (95% CI)	P-trend A	P-trend B	P-trend C
GDQS score							
	T1 (Ref.)	1.00	1.00	1.00			
	T2	0.60 (0.35–1.02)	0.62 (0.36–1.07)	0.68 (0.39–1.18)	**<0.001**	**<0.001**	**0.002**
	T3	**0.30 (0.18–0.52)**	**0.32 (0.19–0.55)**	**0.39 (0.23–0.68)**			
*Per 1-SD*		**0.59 (0.46–0.76)**	**0.61 (0.47–0.79)**	**0.65 (0.50–0.85)**			
Positive GDQS							
	T1 (Ref.)	1.00	1.00	1.00			
	T2	0.58 (0.34–1.00)	0.59 (0.34–1.02)	0.61 (0.35–1.07)	**<0.001**	**<0.001**	**<0.001**
	T3	**0.26 (0.15–0.45)**	**0.27 (0.16–0.47)**	**0.32 (0.18–0.56)**			
*Per 1-SD*		**0.55 (0.43–0.71)**	**0.57 (0.44–0.74)**	**0.60 (0.45–0.80)**			
Negative GDQS							
	T1 (Ref.)	1.00	1.00	1.00			
	T2	1.05 (0.61–1.82)	1.10 (0.63–1.92)	1.22 (0.69–2.16)	0.256	0.355	0.632
	T3	0.62 (0.38–1.02)	0.65 (0.39–1.07)	0.73 (0.43–1.23)			
*Per 1-SD*		0.87 (0.68–1.11)	0.89 (0.69–1.14)	0.94 (0.72–1.22)			
PDQS score							
	T1 (Ref.)	1.00	1.00	1.00			
	T2	0.61 (0.36–1.05)	0.64 (0.37–1.11)	0.67 (0.38–1.18)	**<0.001**	**<0.001**	**0.001**
	T3	**0.27 (0.16–0.47)**	**0.29 (0.17–0.51)**	**0.32 (0.18–0.57)**			
*Per 1-SD*		**0.58 (0.44–0.76)**	**0.60 (0.46–0.79)**	**0.62 (0.47–0.82)**			
Healthy PDQS							
	T1 (Ref.)	1.00	1.00	1.00			
	T2	1.25 (0.72–2.16)	1.28 (0.74–2.22)	1.31 (0.75–2.30)	**0.007**	**0.010**	**0.021**
	T3	**0.46 (0.28–0.75)**	**0.48 (0.29–0.78)**	**0.52 (0.32–0.86)**			
*Per 1-SD*		**0.70 (0.56–0.87)**	**0.71 (0.57–0.89)**	**0.74 (0.59–0.93)**			
Unhealthy PDQS							
	T1 (Ref.)	1.00	1.00	1.00			
	T2	0.72 (0.43–1.21)	0.75 (0.45–1.26)	0.80 (0.47–1.36)	**0.020**	**0.035**	0.097
	T3	0.58 (0.35–0.96)	0.61 (0.37–1.01)	0.67 (0.40–1.12)			
*Per 1-SD*		0.82 (0.67–1.01)	0.84 (0.68–1.03)	0.87 (0.71–1.08)			

OR, odds ratio; CI, confidence interval; T, tertile; MAFLD, metabolic-associated fatty liver disease; GDQS, global diet quality score; PDQS, prime diet quality score; SD, standard deviation.

Model A (Minimally Adjusted): Adjusted for age (years), sex, and total energy intake (kcal/day).

Model B (Core Confounder Adjusted): Model A + physical activity (MET/h/day), familial history of MAFLD (yes/no), and smoking history (yes/no).

Model C (Sensitivity Analysis): Model B + BMI (kg/m^2^), fat intake (g/day), and fiber intake (g/day). Significant values (*P* < 0.05) are presented in bold.

## Discussion

This study demonstrates that higher diet quality, as measured by the Global Diet Quality Score (GDQS) and Prime Diet Quality Score (PDQS), is associated with a reduced likelihood of MAFLD, highlighting the protective role of balanced dietary patterns (6). Previous research has established that dietary patterns, such as the Mediterranean diet, are linked to lower MAFLD risk by modulating the metabolic and inflammatory pathways (2). The observed association may be attributed to the anti-inflammatory and antioxidant properties of nutrient-dense diets, which reduce hepatic fat accumulation and oxidative stress. Our findings contribute to the growing evidence that standardized diet quality scores can serve as reliable tools for assessing MAFLD risk across populations (18).

Building on these findings, specific dietary components, such as increased consumption of fruits, vegetables, and legumes, likely drive the protective effects of higher GDQS and PDQS. While GDQS and PDQS have been validated for assessing diet quality in relation to non-communicable diseases, such as type 2 diabetes and cardiovascular disease, their application to MAFLD remains limited, making our findings a novel contribution (18–20). These food groups are rich in fiber, antioxidants, and polyphenols, which may modulate the gut–liver axis, reducing inflammation and improving lipid metabolism (21). This finding suggests that dietary interventions emphasizing these foods could be effective in MAFLD prevention, particularly in regions with high processed food consumption.

The lower consumption of refined grains and sugar-sweetened beverages in controls compared to cases suggests that reducing processed food intake could be a practical strategy for MAFLD prevention. Studies have shown that high intake of refined carbohydrates and added sugars exacerbates insulin resistance and hepatic steatosis, key drivers of MAFLD (22). The differences observed in our study may reflect dietary habits that promote metabolic dysfunction in cases, which may be potentially exacerbated by low physical activity or genetic predispositions. These findings underscore the need for public health campaigns to reduce processed food consumption as part of MAFLD prevention strategies.

In the context of Al-Kharj, Saudi Arabia, our findings highlight the relevance of dietary interventions tailored to local dietary patterns. The high consumption of refined grains in cases may reflect cultural preferences for processed foods, which are increasingly prevalent in the region. This regional dietary pattern likely contributes to the elevated MAFLD prevalence observed, suggesting that culturally sensitive interventions could enhance dietary adherence. Future studies should explore the scalability of GDQS and PDQS in diverse populations to confirm their utility in global MAFLD prevention efforts.

The notably higher legume consumption among controls compared to cases suggests that legumes may play a protective role against MAFLD (23). Research indicates that legumes, rich in dietary fiber and plant-based proteins, contribute to improved metabolic health and reduced liver fat accumulation (24). This difference may be explained by the role of legume-derived fiber in promoting satiety, stabilizing blood glucose levels, and fostering a healthy gut microbiota, which mitigates inflammation via the gut–liver axis. Incorporating legumes into dietary guidelines could enhance MAFLD prevention strategies, particularly in populations with low legume intake.

Furthermore, the higher intake of fish and shellfish among controls compared to cases points to the potential benefits of omega-3 fatty acids in reducing the MAFLD risk (25). Studies suggest that omega-3 fatty acids, which are abundant in fish, have anti-inflammatory properties and improve lipid profiles, which may counteract hepatic steatosis (26). The observed difference likely stems from the ability of omega-3 s to modulate lipid metabolism and reduce pro-inflammatory cytokines, key factors in MAFLD progression. These findings advocate for increased fish consumption as part of a balanced diet to lower MAFLD prevalence, especially in regions with limited seafood intake.

The elevated consumption of processed meats in cases compared to controls highlights the detrimental impact of these foods on MAFLD risk (27). Processed meats, high in saturated fats and additives, have been linked to increased insulin resistance and hepatic inflammation (28). This dietary pattern may exacerbate MAFLD by promoting oxidative stress and dyslipidemia, which are central to disease pathogenesis. Reducing processed meat intake should be prioritized in dietary interventions to mitigate MAFLD risk, particularly in populations with high consumption of processed foods.

This study has several strengths, including a clear focus on the association between diet quality and MAFLD, the use of validated dietary indices (Global and Prime Diet Quality Scores), and a case–control design that allows for direct comparison between affected and unaffected individuals. Additionally, focusing on MAFLD—a prevalent metabolic disorder—enhances the clinical relevance of the findings. However, there are some limitations to consider. The case–control design prevents causal inference, and dietary data based on self-reported questionnaires may be subject to recall bias. The generalizability of the results may be limited to the studied population, and residual confounding from factors such as genetics, physical activity, or comorbidities cannot be entirely ruled out. Finally, more detailed reporting on the calculation and interpretation of the dietary scores would strengthen the clarity and reproducibility of the findings.

## Conclusion

Our findings underscore the potential of high-quality diets, as assessed by GDQS and PDQS, to mitigate the global burden of MAFLD. By promoting diets rich in nutrient-dense foods and low in processed items, clinicians and policymakers can develop targeted interventions to prevent MAFLD, particularly in high-risk populations such as those in Al-Kharj, Saudi Arabia. These results pave the way for integrating standardized diet quality scores into routine clinical practice and public health strategies, offering a practical approach to reducing liver disease prevalence.

## Data Availability

The original contributions presented in the study are included in the article/supplementary material, further inquiries can be directed to the corresponding author.
